# Temperature Sensing Optimization for Home Thermostat Retrofit

**DOI:** 10.3390/s21113685

**Published:** 2021-05-26

**Authors:** Federico Seri, Marco Arnesano, Marcus Martin Keane, Gian Marco Revel

**Affiliations:** 1 College of Science and Engineering, National University of Ireland, Galway, Ireland; marcus.keane@nuigalway.ie; 2 Informatics Research Unit for Sustainable Engineering (IRUSE), Galway, Ireland; 3 Ryan Institute, National University of Ireland, Galway, Ireland; 4 Università Telematica eCampus, 22060 Novedrate, Italy; marco.arnesano@uniecampus.it; 5 Department of Industrial Engineering and Mathematical Sciences, Università Politecnica delle Marche, 60131 Ancona, Italy; gm.revel@univpm.it

**Keywords:** sensing optimization, thermostat retrofit, building simulation, optimization, thermal comfort, smart thermostat, thermostat placement

## Abstract

Most existing residential buildings adopt one single-zone thermostat to control the heating of rooms with different thermal conditions. This solution often provides poor thermal comfort and inefficient use of energy. The current market proposes smart thermostats and thermostatic radiator valves (TRVs) as cheap and relatively easy-to-install retrofit solutions. These systems provide increased freedom of installation, due to the use of wireless communication; however, the uncertainty of the measured air temperature, considering the thermostat placement, could impact the final heating performance. This paper presents a sensing optimization approach for a home thermostat, in order to determine the optimal retrofit configuration to reduce the sensing uncertainty, thus achieving the required comfort level and minimizing the retrofit’s payback period. The methodology was applied to a real case study—a dwelling located in Italy. The measured data and a simulation model were used to create different retrofit scenarios. Among these, the optimal scenario was achieved through thermostat repositioning and a setpoint of 21 °C, without the use of TRVs. Such optimization provided an improvement of control performance due to sensor location, with consequent energy savings of 7% (compared to the baseline). The resulting payback period ranged from two and a half years to less than a year, depending on impact of the embedded smart thermostat algorithms.

## 1. Introduction

According to [1], the stock of residential buildings in the EU is relatively old, with more than 40% having been built before 1960 and 90% before 1990. Older buildings typically use more energy than new ones. The rate at which new buildings either replace this old stock, or expand the total stock, is low (about 1% a year). This implies that the reduction of energy consumption of buildings should not exclude the renovation of existing buildings. However, the renovation rate is also low, with only about 1–2% of the building stock being renovated each year [2]. Similarly, the residential building stock is subject to a long renovation cycle, according to [3].

Considering the age of the European building stock, most dwellings have changed their partition design and room usage over the years. Moreover, the construction materials initially used have degraded, thus decreasing thermal performances. The heating equipment have also become obsolete, leading to discomfort and higher energy consumption than necessary. Increasing the performance of buildings is the only solution to solve the problem of existing buildings that are not comfortable, either affecting the well-being and health of the occupants or driving them to take actions that may compromise the energy economy of the building, as has been confirmed in [4]. Global climate targets and future building consumption levels cannot be reached by only improving insulation or installing advanced heating/cooling system technologies, according to [5]. In addition, the IEA has estimated that 53% of household final energy consumption can be attributed to space heating [6,7].

Considering the age of the European building stock, its low renovation rate, the low level of indoor thermal comfort provided to inhabitants, and the magnitude of energy consumption due to indoor space heating, building retrofits focused on heating/cooling control systems could provide an important contribution. 

The current market is proposing new, cheap, and relatively easy-to-install solutions to retrofit existing heating system controls. In particular, new smart thermostats [8,9,10,11] offer the possibility to replace existing ones, both in terms of position and heating control. Among the proposed solutions, the most adopted one concerns a system composed of a wireless temperature sensor that can be installed in any room, connected by a radio signal to a relay which, replacing the existing thermostat, is wired to the boiler to control its operation. There is also the possibility to extend the monitoring system by adding thermostatic radiator valves (TRVs) to control the heating/cooling sources in a smarter way, optimizing the thermal load distribution within the dwelling. In particular, [12] analyzed the application of thermostatic TRVs to an old existing multi-family building in Italy; the study demonstrated that the application of dynamic energy simulation to different patterns of TRV use brought significant energy savings, from a minimum of 2% up to a maximum of 10%. Meanwhile, in [13], long-term field data were collected over several heating seasons, from nine existing multi-family residential buildings in Poland equipped with TRVs. The energy savings ranged between 7.1% and 23.3%, and the payback time was less than 2.5 heating seasons in all cases.

Such new programmable thermostats also include evolved heating control approaches: from reaching and maintaining a pre-defined temperature setpoint, to shifting setpoints during times when lower temperature levels are reasonable and incorporating control-loop external influences (e.g., weather compensation). Moreover, they can integrate advanced control algorithms and can lead to significant energy savings. One study [14] aimed to identify the impact of thermostat strategies on heating and cooling energy consumptions in residential buildings. They concluded that the setpoint temperature of thermostat had a significant impact on the heating and cooling energy consumptions, and the setback temperature during the nighttime setback showed non-significant energy savings, compared to the setpoint temperature variation. Another study [15] collected data on room temperature, heating behavior, and occupancy patterns of households in Southern Germany over a 14-month period. They found that temperature setpoint variation could lead to median savings potentials in the range of 21–26% and observed higher thermal comfort, compared to programmable thermostats. The reported results also suggested that the focus of policy should extend from retrofitting heating systems and building insulation towards more efficient energy use enabled by intelligent control.

However, considering the study presented in [16], which evaluated the impact of thermostat usability on facilitating the energy-saving behaviours of thermostat users, something went wrong. The reported study reached the following conclusions: 1. “High usability thermostat was not sufficiently easy to use;” and 2. “Study participants adjusted their thermostats to override the default schedule. Regardless of the thermostat model, they managed to keep the temperature at the level that ensured their comfort and negated any energy saving features.” In [17], an extensive literature review regarding surveys on the impact of smart thermostats in residential buildings was reported; the authors concluded that smart thermostat systems are, in the short-term, a promising low-investment option for households in buildings with low to medium efficiency standard, which are not expected to be energetically refurbished in the coming years; however, in the long run, the thermal insulation of buildings is indispensable, and more comprehensive energy management approaches—at least at the building level—seem to offer greater potential for reducing both energy consumption and CO_2_ emissions. Meanwhile, Ref. [18] found that smart thermostats are becoming prevalent in residential buildings; however, occupant usage of these devices is not well-understood, compared to conventional programmable thermostats. In particular, a survey and thermostat data from 54 suites were used to characterize smart thermostat programming and usage behaviours, alongside self-reported environmental values and technical skill levels. Based on this characterization, several opportunities and challenges for reducing energy use through the use of smart thermostats were identified. Adjusting default thermostat settings—in particular, default schedules and default override behaviours—is recommended to promote energy-conscious thermostat usage behaviours. 

In addition, the lack of positive results from the adoption of smart thermostats could become even worse, if the problem of measuring the air temperature is not correctly addressed. 

In residential buildings, the heating system should be designed and operated to reach the stated requirements in relation to the environment, comfort, and operating economy. The control of multiple rooms is commonly performed with a single-zone thermostat measuring the air temperature in one room of the dwelling, without considering the air temperature differences in the adjacent rooms. For this reason, the thermal comfort is not satisfied in all rooms at the same time, but mainly in the controlled one, and heating energy is consumed inefficiently. In fact, descriptions of the criteria adopted to design the control system, concerning the placement of the thermostat, are often omitted, as it is generally based on experience and without considering the importance of sensing the air temperature at the right location in the dwelling. Previous works have partially addressed this reported issue. In particular, in [19], the authors proposed a framework based on a coupled CFD (computational fluid dynamics)–BES (building energy simulation) simulation model, in order to optimize HVAC systems with non-uniform airflow and temperature distributions in the building design, to achieve good thermal comfort and energy efficiency. The optimization platform was demonstrated to search for the optimal thermostat placement in an office room with displacement ventilation and a VAV terminal box. A similar approach has been applied to large sport facilities in [20]. In any case, the previous works have been focused on non-residential buildings and large spaces, where the air temperature distribution can be considered non-uniform. 

Considering the literature review reported in the previous paragraphs, the following outcomes can be recognized:

- Heating or cooling strategies based on temperature setpoint variation could lead to energy savings and thermal comfort improvement in residential buildings;
- A smart thermostat alone cannot lead to the expected improvement, in terms of energy savings and thermal comfort; and
- Advanced studies on indoor air temperature measurement accuracy and thermostat placement have focused mostly on large spaces, not in residential buildings.

A study that combines smart thermostat placement and usage to optimize thermal comfort and energy consumption in residential buildings has been found in the literature. In particular, Ref. [21] developed a methodology to find the optimal configuration of a multi-sensor monitoring system to decrease the energy consumption and match the comfort conditions within the ASHRAE comfort zone. However, this study was suitable for buildings with HVAC systems, excluding the cases with traditional heating systems based on radiators. 

Considering the outcomes of the literature review, this paper presents an innovative methodology, which is specific to residential buildings, in order to optimally configure the heating retrofit, taking into account the measurement uncertainty due to thermostat placement, thermal comfort, advanced heating strategies, and costs. In particular, we investigate the problem of measuring the air temperature in the optimal location of the building with the optimal setpoint, considering the possibility of additionally installing TRVs. The proposed approach is based on building simulation modelling, coupled with criteria for guaranteeing thermal comfort with a reasonable payback period.

The paper is organized as follows: A description of the methodology, the modelling approach, and selection of optimization criteria are presented in the following section. Then, the case study is presented, highlighting the application of the proposed methodology. The results of the case study are further illustrated through a discussion on the impact on energy savings. 

## 2. Methodology

This study proposes a methodology based on dynamic building simulation, in order to optimize the retrofitting of an existing heating control system for residential buildings, taking into account the optimal location for measuring the air temperature to reduce control errors due to uncertainty. In particular, the methodology provides, as output, the optimal configuration of the heating system, in terms of:

1. Air temperature sensing strategy (thermostat location).
2. Optimal air temperature setpoint to be used during the occupied hours.

The optimization outcome is based on two KPIs—one for thermal comfort and one for payback period—evaluated for the “as-is” scenario (before retrofit) and “what-if” scenarios, generated with the support of dynamic simulations.

### 2.1. Building Modelling 

**Figure 1:**
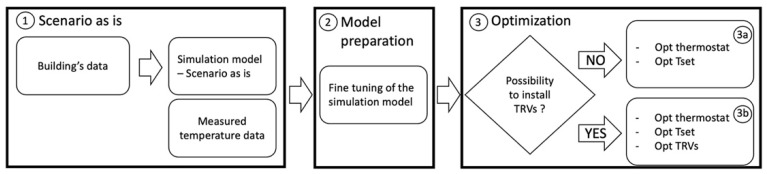
Workflow of the proposed methodology.

The proposed approach is based on a white box for the dynamic simulation model of the dwelling, which was used to analyse the current heating performance (Figure 1; “as-is” scenario, step 1) and to create retrofit scenarios. The white box model approach is law-driven and applies physical laws to predict the system behaviour. White box models are highly detailed and can be used for operational analysis through calibration [22]. Due to their level of detail, white box models represent building dynamics and performance extremely well. The model inputs range from external weather conditions, building descriptions, building fabric information, and building energy system description. These inputs are collected through documents retrieved at various stages of the building life cycle, such as the design stage, the as-built stage, and the operation stage. The simulation engine consists of detailed calculations and mathematical algorithms, which involve thermal load calculations, system simulation, and plant analysis. Various white box model tools which provide a basis for the simulation engine are available, such as Energy plus [23], TRANSYS [24], and IESVE [25]. 

The simulation model was developed using the IESVE software [25], which is an in-depth suite of integrated analysis tools providing high-quality simulation-based information, which is required to design, build, and operate better-performing sustainable buildings which maintain the required comfort levels. For this study, several software modules were used. Specifically, the ModelIT allows for the generation of geometry of the building simulation model; ApacheSim generates the thermal simulation model for the building; MacroFlo is used for analysing infiltration and natural ventilation; ApacheHVAC simulates the heating/cooling control system in a detailed way, allowing for the evaluation of a proposed retrofitting solution; and VE-script is a Python environment for task automation, providing add-on functionality such as post-processing analysis of the results. VE-script was used to evaluate indoor thermal comfort levels and energy savings.

To achieve the required accuracy, the simulation model was fine-tuned using IoT temperature sensors installed in each room of the dwelling (Figure 1, step 1). The monitoring should be performed for a minimum period of two weeks inside the dwelling, during the heating season. In this way, the thermal characteristics of the house, related to the heating systems and behaviour of occupants, can be captured and used for model fine-tuning. Once the data are collected, a simulation model of the dwelling, replicating the “as-is” building’s performance (Figure 1, step 2), is generated. Trends of air temperature in each room are finally generated. A detailed description of the simulation model is reported in Section 3.2, while the following section describes the KPIs utilized during the optimization process. 

### 2.2. KPIs for Optimization

The selection of the optimal sensing configuration was based on two main KPIs: thermal comfort and heating energy cost.

According to EN 16798 [26], thermal comfort can be assessed using predictive and adaptive comfort models. The latter is considered more suitable for naturally ventilated buildings, while the former is more suitable for buildings with mechanical ventilation. In both cases, the building performance can be assessed using long period indicators, calculated as the percentage of time during which the building operates outside the comfort limits. Considering residential buildings, occupants have easy access to operable windows and can freely adapt their clothing to the thermal conditions, while the thermal response differs from that of occupants of buildings with HVAC systems and depends, in part, on the outdoor climate. The indoor operative temperatures are compared to the external running mean temperature, and the difference is assessed against a set limit to identify the time outside an acceptable comfort range. Thus, the thermal comfort KPI used in this study was defined as *POR*: the percentage of time during which the building operated outside the comfort limits. For each room, the hourly operative temperature *T_o_* was extracted from the simulation model and, for each day, the outdoor running mean temperature, *T_rm_*, is calculated as:

(1) $$T_{rm,j}=\frac{T_{ed-1}+0.8T_{ed-2}+0.6T_{ed-3}+0.5T_{ed-4}+0.4T_{ed-5}+0.3T_{ed-6}+0.2T_{ed-7}}{3.8}\;\left[{}^{\circ}C\right],$$

where *T_ed_*_−1_ is the daily average of hourly external temperatures for the previous day (according to location weather data) and *T*_*ed*−2…7_ are the daily averages of hourly external temperatures for the 2nd–7th prior days (according to weather data). For every room and every hour, the difference between the operative and running mean temperature (Δ*T_i_*) is calculated as follows:

(2) $$\Delta T_{i}=T_{o,i}-0.33T_{rm,j}-18.8\;\left[{}^{\circ}C\right].$$

For every room the number of occupied hours outside the range (*h_or,i_*) was calculated as the number of occupied hours when |Δ*Ti*| ≥ |Δ*T_lim_*|. Δ*T_lim_*, in this study, was set to ±3 °C, according to the EN 16798 classification criteria. Thus, the percentage of occupied hours outside the range, ($POR_{i}$) [%], is calculated as follows:

(3) $$POR_{i}=\frac{h_{or,i}}{h_{tot}}*100\;\left[\%\right].$$

The *POR* of the entire dwelling is finally calculated as the average of the *POR_i_* for each room, weighted according to the floor area:

(4) $$POR=\frac{\sum_{i=1}^{n}POR_{i}*S_{i}}{\sum_{i=1}^{n}S_{i}}\;\left[\%\right],$$

where *n* is the number of rooms and *S_i_* is the floor area of the *i*th room.

The *POR* was used as performance criterion to evaluate the building performance, in terms of thermal comfort, before and after the retrofit intervention. A *POR* equal or lower than 5% of the occupied hours was considered acceptable. The analysis was performed only considering the main occupied rooms (e.g., bedrooms, living room, kitchen), and did not consider short-term occupancy and transit areas (e.g., bathrooms, corridors, small storage areas).

The second criterion for the selection of the optimal retrofit solution was based on a simplified payback period (*PB*). This KPI, which is related to the cost analysis, is calculated as:

(5) $$PB=\frac{C_{retrofit}}{\left(HC_{baseline,\;year}-HC_{retrofit,\;year}\right)}\left[y\right],$$

where *C_retrofit_* is the cost of the retrofit solution (smart thermostat and TRVs), *HC_baseline,year_* is the yearly heating energy cost before the retrofit, and *HC_retrofit,year_* is the yearly heating energy cost after the retrofit. The lower the *PB*, the more convenient the retrofit.

### 2.3. Optimization for Home Thermostat Retrofit 

Once the simulation model is calibrated, the methodology proceeds with the optimization step (Figure 1). Depending on the typology of the heating system (i.e., possibility to install TRVs), two optimization processes are proposed. In the case that there is no possibility to install thermostatic valves on the radiators, an optimization process to find the optimal placement of the thermostat with the optimal setpoint is proposed (Figure 1, point 3a). 

First of all, the as-is scenario evaluates the thermal comfort KPI in the current state of the building. If the thermal comfort criterion is not satisfied, the optimization process continues running a what-if scenario, consisting of modifying the existing thermostat setpoint until the *POR* achieves the minimum requirement (≤5%), where all rooms are in comfort conditions. This what-if scenario is considered as baseline for the further retrofit optimization steps. It represents the existing configuration of the heating control system, using the optimal setpoint to guarantee the thermal comfort inside the dwelling. The energy consumption calculated for this scenario is used as a baseline for the calculation of *PB*, which is used to determine the optimal solution.

Once the Optimal setpoint is defined, new what-if scenarios are generated. Each scenario consists of placing the new thermostat in a different room, covering all the rooms of the dwelling (except for bathroom and store places) with temperature setpoints from 19–24 °C, with steps of 1 °C. Therefore, the optimization runs a number of simulations considering the combinations of thermostat placements and temperature setpoints. 

The *POR* is evaluated for all the previous scenarios. The scenarios that satisfied the *POR* are those selected for analysis, in terms of *PB*.

The cost analysis defines the optimal thermostat placement configuration, in terms of the payback period. Finally, the what-if scenarios that satisfied the *POR* are selected to determine the optimal heating system configuration; that is, the optimal thermostat placement and final optimal setpoint.

In the case that there is also the possibility to install TRVs, the optimization procedure continues generating a what-if scenario with a TRV in each room. In this case, the new thermostat is placed in the original position and the terminals located in the other rooms (except the one where the thermostat is installed) are equipped with thermostatic valves. Then, the temperature setpoint is adjusted (temperature setpoints from 19–24 °C, with steps of 1 °C) to reach the best satisfaction of the *POR* criterion. Once it is satisfied, the calculated setpoint is considered as the Optimal Tset. Then, the *PB* is calculated, in order to identify the optimal solution, in comparison with the result coming from the previous optimization process.

## 3. Test Case

The test case selected for the application of the methodology was a residential building located in Jesi (AN), Italy. In particular, the analysis was performed for a dwelling on the second floor of the building. The following sections illustrate the building data collection, simulation model development, sensor network installation for model calibration, and optimization process, and, finally, a discussion of the results is provided.

### 3.1. Description of the Case Study

The dwelling under analysis consisted of a kitchen, a living room, two bedrooms, and two bathrooms. The heating system was composed of a single non-condensing boiler with heat capacity of 23 kW and 6 radiators. The thermostat, placed inside the kitchen, provided the air temperature value measured in that zone as feedback to the boiler. The thermostat was programmed to switch on the heating system from 7 to 9 a.m. and from 6 to 11 p.m. There was no cooling system. The building characteristics essential to the analysis are presented in Table 1.

### 3.2. Mesurements for Model Fine-Tuning

**Figure 2:**
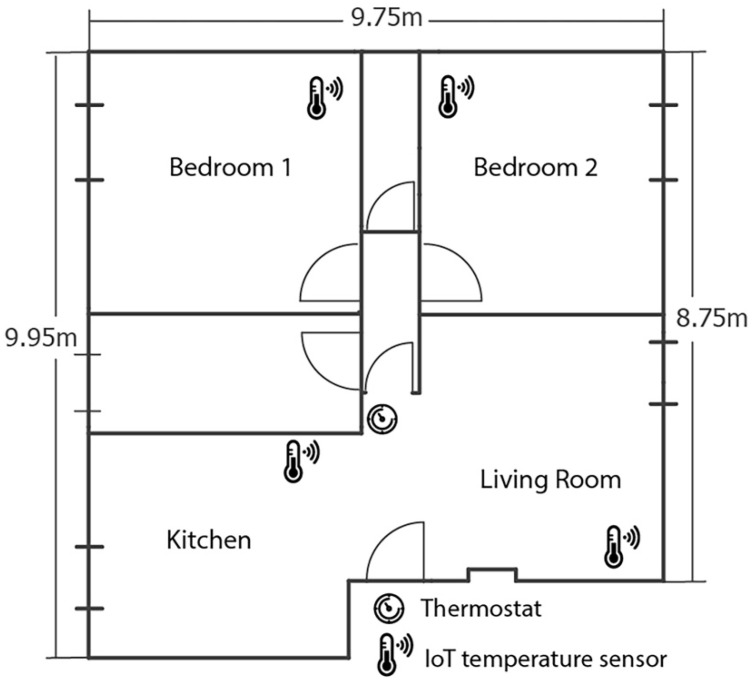
Layout of the dwelling with the location of the existing thermostat and sensors used for the measurement campaign.

A sensor network was installed to capture the thermal behaviour of the dwelling under analysis. The monitoring system was composed of four air temperature sensors, placed inside the kitchen, living room, bedroom 1, and bedroom 2 (Figure 2), respectively. 

**Figure 3:**
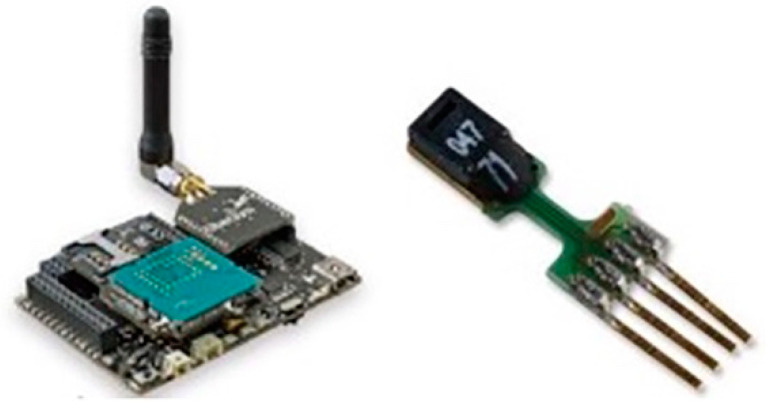
Waspmote board and Sensirion SHT75.

The measurement campaign was performed during the second half of December. The IoT sensor network was made using four Waspmote boards, equipped with Sensirion SHT75 (accuracy ±0.4 °C, resolution 0.1 °C) sensors, as shown in Figure 3. The sensors were mounted on the perimetral walls at 1.7 m from the ground, the same height as the existing thermostat. Sensor locations with the presence of large windowed surfaces, direct solar radiation, air drift, or corners with possible air stagnation phenomena are not suitable for sensor installation. 

**Figure 4:**
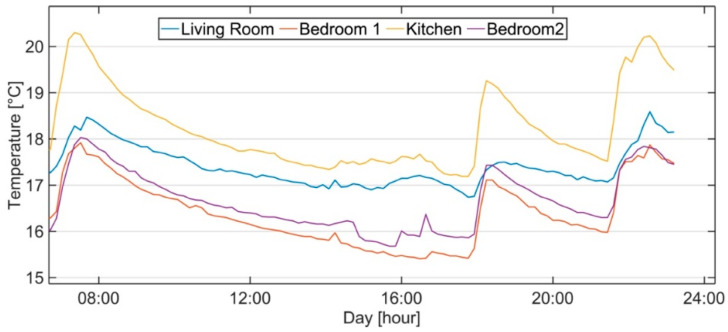
Indoor air temperature profiles measured during a typical winter day.

Figure 4 shows one day’s temperature profiles for each room measured during the campaign. The heating system was configured using the thermostat located in the kitchen (Figure 2), at a setpoint of 20 °C, which was scheduled to work between 07:00–9:00 a.m. and 06:00–11:00 p.m., providing thermal loads to the different rooms. The boiler stopped working around 8 a.m., when the setpoint temperature was reached inside the kitchen. The dead-band of 1 °C did not allow the system to restart before the 9 a.m. Thus, during those two hours, the other rooms were under-heated, with a gradient of 2 °C compared to the kitchen air temperature.

During the afternoon, the thermostat started working again, with a setpoint of 19 °C at 6 p.m., and the boiler stopped around 7 p.m. At 9 p.m., the heating system was active again until 11 p.m., with a setpoint of 20 °C. In the evening, the gradients remained constant and the condition of under-heating for the other rooms continued. 

**Figure 5:**
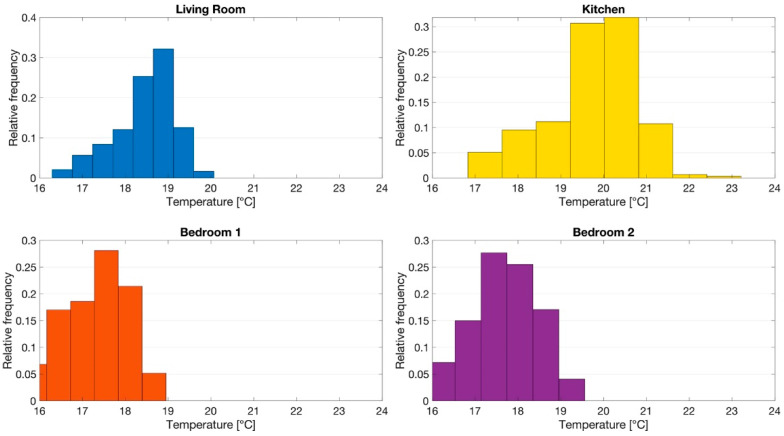
Relative frequencies of air temperature measured in each room.

The monitoring task pointed out consistent air temperature gradients between rooms, as confirmed by the relative frequencies shown in Figure 5. The thermostat located in the kitchen was able to provide and maintain the selected setpoint for the room, but the other rooms remained, most of the time, in under-heating conditions. 

Results from the monitoring campaign underlined the importance of a retrofit measure, in order to overcome thermal comfort issues due to the inhomogeneous temperature distribution.

### 3.3. Optimization of the Sensing System

The simulation model was developed based on the data reported in Table 1. The challenge here was to generate a simulation model that was able to reproduce the indoor thermal comfort inside the different rooms of the apartment, allowing the optimization process to define the best retrofit scenario for the thermostat. Particular attention was paid to the heating system sizing and control loop. In Apache HVAC, two separate environments allow for simulation of the air and water parts of the heating system; in our case, only natural ventilation was present. For each room, the characterization of the heating terminals and control topology had to be set up, as well as internal gains and infiltrations, which were the main parameters used to refine the simulation model performance.

Using the measured data, the simulation model was fine-tuned to reproduce the real thermal behaviour of the building. It should be considered that the simulation model uses generic weather data at the building’s location, and not the real data. This could lead to an incorrect comparison between real indoor temperature trends and simulated ones. In particular, considering the campaign period, a comparison based on heating degree days (HDD) allowed for the selection of the most similar day between the real outdoor air temperature trend and the simulated one, as provided by IESVE software, following an approach similar to the work presented in [27]. The approach can be split into three steps: 1. Determining the Base Temperature; 2. extraction of HDD from a local weather station and the weather simulation model file; and 3. selection of the most representative day between those available for the calibration, considering the minimum deviation between the real and simulated HDDs.

**Figure 6:**
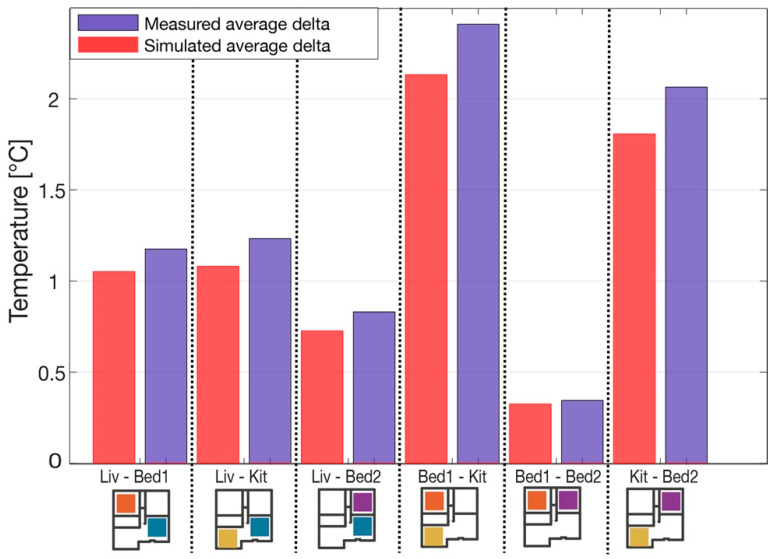
Comparison of simulated vs. measured air temperature gradients between rooms.

The objective of the calibration was to reproduce the indoor air temperature inhomogeneity within the different rooms of the dwelling, as reported in Figure 4. Therefore, the model tuning was based on minimizing the difference in the absolute values of temperature gradients (simulated versus measured data) between rooms on the selected day. This allowed for replication of the thermal behaviours of the different rooms reacting to different weather conditions. For a clear measure of the model performance, a comparison based on air temperature gradients between rooms is reported in Figure 6.

**Figure 7:**
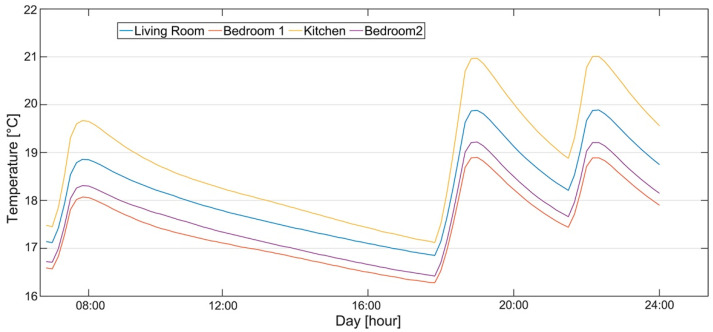
Indoor air temperature profiles measured during a typical winter day.

The calibration process was performed manually, by using a radiator adjustment function, as well as modifying infiltration and internal gains. The final model turned out to provide a maximum deviance of 10% between the measured and simulated data (Bedroom 1 versus Kitchen) for the selected day. Figure 7 reports the simulated air temperature trends after model calibration. 

As shown in Figure 7, the simulation model followed the temperature fluctuations during the day well, according to the indoor air temperature trends reported in Figure 4.

The optimization methodology, detailed in the previous sections, was applied to this test case. First, the as-is scenario was developed, with the thermostat placed in the kitchen having a temperature setpoint of 20 °C. The POR index was equal to 35%, which indicated a high percentage of time during which the building operated outside the comfort range. As it overcame the limit for thermal comfort satisfaction, the as-is scenario was adjusted to create a baseline where the POR limit was respected.

Then, all the what-if scenarios were analysed by moving the thermostat location from one room to another and performing the POR analysis. The best configuration had the thermostat located in Bedroom 2 with a setpoint of 21 °C. As the heating system allowed for the installation of thermostatic valves, the optimization process continued developing the what-if scenarios with TRVs. The Tset, when increased by 1 °C, satisfied the POR analysis; therefore, the Optimal Tset was defined as 21 °C. The selected best solutions (i.e., those which were able to satisfy the POR) were then evaluated, in terms of PB, for identification of the optimal solution. For the proposed case study, an average cost of 165 € was considered for an off-the-shelf smart thermostat, and 70 € for each TRV. Energy costs were calculated considering the cost of natural gas, which was used to operate the boiler, being equal to 0.65 €/Nm^3^. Table 2 summarizes the results of the retrofit process, including the cost analysis.

The as-is scenario was simulated, showing a TOT POR of 35% and a heating consumption of about 530 Nm^3^/y, which had a deviation of 7.5%, with respect to the real gas consumption reported from bills (490 Nm^3^/y). The what-if scenario, with the old thermostat position and setpoint raised to 24 °C, kept the dwelling under thermal comfort condition at a cost of 554 € per year (baseline for the cost analysis). This scenario presented the highest heating consumption, compared to the other retrofit solutions, due to the overheating conditions generated in the kitchen to achieve comfortable temperatures in the other rooms. The what-if scenario with TRVs reduced the heating consumption to 717 Nm^3^/y and energy cost to 466 €, with a retrofit expenditure of 375 € and a POR of 3.7%. The best solution (bold highlighted in Table 2) was that using the new thermostat placed in the bedroom 2 with a setpoint of 21 °C, satisfying the comfort requirements (POR equal to 4%). The heating consumption was raised to 745 Nm^3^/y—slightly higher, with respect to the scenario with TRVs. However, this solution, with a heating energy cost of 484 € and retrofit cost of 165 €, led to a payback period of 2.4 years, demonstrating the effectiveness of the proposed solution, which was financially more attractive than that using TRVs.

### 3.4. Uncertainty Due to Thermostat Placement

As shown in previous sections, the accuracy of the thermostat operation could be affected by measurement deviation due to the installation point. In fact, the experiment conducted in the real case study showed a significant difference between the air temperature measured in each room (Figure 4). So, if the desired measurand is a temperature representative of the overall thermal condition, each sensing location is characterized—among the others—by an uncertainty component, which could be quantified as the deviation from a reference condition, represented by the temperature (*T_r_*) calculated as the average of measured room temperatures (*T_i_*) weighted by the floor area (*A_i_*):

(6) $$T_{r}\left(t\right)=\frac{\sum_{i}^{n}T_{i}\left(t\right)\cdot A_{i}}{\sum_{i}^{n}A_{i}}.$$

The data acquired during the monitoring campaign were used to calculate the reference time-series, *Tr*, to be compared with the baseline scenario (kitchen) and with the optimal solution (bedroom 2). Table 3 recaps the statistics of the three time-series.

To estimate the measurement accuracy due to the installation point, the deviation between *T_r_* and *T_kitchen_* and the deviation between *T_r_* and *T_bedroom_*_2_ were calculated. The thermostat installed in the kitchen turned out to provide a deviation (mean and std) of 1.5 ± 0.4 °C, with respect to the reference *T_r_*. Meanwhile, the thermostat installed in bedroom 2, according to the optimization results, decreased the deviation to 0.6 ± 0.3 °C. This result confirms that reducing the uncertainty due to thermostat placement can provide more efficient control of the heating system.

## 4. Discussion

The application to the case study turned out to prove that the best retrofit solution consisted of the selection of a new sensor location for the thermostat operation. This result confirms that the measurement of the air temperature for a single-zone feedback loop plays a pivotal role. Common strategies, such as measuring the temperature in the worse-case room or averaging the hottest and coldest rooms, cannot guarantee the required level of comfort in each room, or may not lead to an efficient use of heating energy. A second thought derived from the case study is that TRVs, in some cases, may not be the most efficient solution, especially when considering the payback period. Even with a non-smart thermostat, air temperature sensor optimization could provide benefits on the same order as the installation of TRVs.

In addition, if the retrofit is carried out using a new smart thermostat, the impact of the increased smartness should be considered. In fact, the retrofit scenario applied to the case study was analysed considering a new thermostat that is capable of simple on/off control with a dead-band of 1 °C. This typology of control was a basic one, but other commercial smart thermostats allow for the use of more sophisticated control algorithms: Compensation with respect to weather forecast, occupancy evaluation, and PID controllers. As reported in [17], the major finding from the field studies was that the average savings provided by a smart thermostat are reasonably well-determined for single-family houses in the U.S., but the factors that influence relative savings are so numerous and the total sample size is so small that quantification can be challenging in other countries. Therefore, in this study, we assumed that the energy savings provided by the smart thermostat could range from 0 to 30%, compared to non-smart thermostats [28]. 

**Figure 8:**
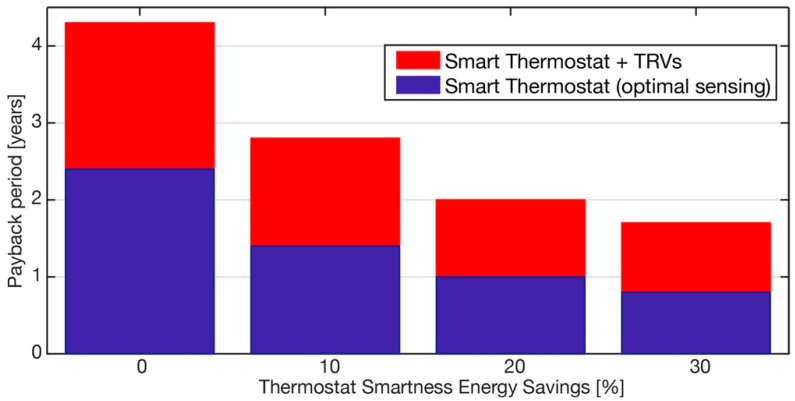
Payback period analysis of proposed retrofit solutions as a function of the energy savings produced by thermostat smartness.

An analysis of the payback time for the retrofit solutions is reported in Figure 8. It compares the baseline scenario with the scenario using a smart thermostat plus TRVs, as well as the scenario with a smart thermostat installed in bedroom 2, according to the optimization results. In particular, the figure reports the payback period of the two solutions, considering the impact of the thermostat smartness providing energy savings in the range 0–30%. 

In the case that the smart algorithms have no effect (point zero on x-axis) on the energy savings, the payback period was the same as that calculated in Table 2. The effectiveness of the smart thermostat could decrease the payback period from four to two and a half years for the what-if scenario All-in, which was aligned with the values reported in [13]. Concerning the best what-if scenario, the payback period decreased from two and a half years to less than one year. 

## 5. Conclusions and Future Work

In this paper, a methodology to optimize the thermostat sensor for the feedback loop in existing heating systems of residential buildings was described, together with its application to a real test case. A simulation model of a dwelling in Italy was developed using the IESVE software, including details related to the heating control system. A sensor network was installed inside the dwelling, in order to measure its air temperature distribution. A significant difference of air temperature was registered between the different rooms, even with the heating switched on. The entire methodology was applied to create baseline and retrofit scenarios. The scenarios were evaluated, in terms of thermal comfort and payback period, to determine the optimal sensing strategy (room for thermostat relocation). This demonstrated that the optimal thermostat placement was in bedroom 2, with an optimal temperature setpoint of 21 °C (no TRV needed). The new optimized sensing configuration for the heating system provided the right level of thermal comfort for tenants and allowed a potential energy savings of 7%, compared to the baseline (i.e., the existent monitoring/control system configuration), with a payback period ranging from two and half years to less than a year, depending on the additional savings provided by the smart thermostat algorithms. The proposed investigation confirmed the importance of the thermostat placement. In particular, the optimal solution provided a reduction of the measurement uncertainty due to the thermostat placement, with the consequent achievement of a properly balanced heating control. Moreover, using the proposed optimization approach, the benefit of using TRVs was also investigated. In the real case study, the optimized thermostat placement turned out to provide benefits comparable to the addition of TRVs, but with a shorter payback period. 

Finally, this study was based on the development and calibration of a building simulation model using a white box approach, which provided the required accuracy for the analysis; however, this could be a limitation, as it is time-consuming, costly, and requires a certain number of detailed inputs. A solution to overcome the mentioned issue is to target the typical typology of dwellings, depending on the country. Therefore, once the simulation model is developed, in terms of the geometry, heating/cooling system, and control loop, the application of the same model and methodology to another similar building would only require small adjustments, justifying the cost of the initial model development. Further studies are needed, regarding the impact of sensor measurement uncertainty, for different commercial thermostats, on the estimation of thermal comfort and energy consumption.

## Figures and Tables

**Table 1:** Characteristics of the case study.

Info	Value	
Building type	Residential	
Dwelling	Second floor	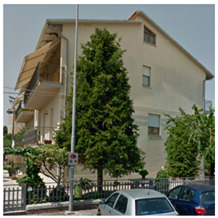
Room usage	Kitchen, Living room, Bedroom 1, Bedroom 2, Bathroom 1, Bathroom 2	
Balcony	Two balconies	
Floor hight	3 m	
Location	Jesi (AN), Italy	
Latitude	43.52° N	
Longitude	13.24° E	
Elevation	24 [m]	
External walls	Brickwork (outer leaf) 180 mm, Glass wool 10 mm, Brickwork (inner leaf) 180 mm, Plasterboard 10 mm	
External windows	Outer pane 4 mm, air cavity 8 mm, inner pane 4 mm	
Internal partitions	Plasterboard 10 mm, common brick 100 mm, plasterboard 10 mm	
Roof	Insulation 20 mm, membrane 2 mm, concrete deck 100 mm, plasterboard 10 mm	
Ground/Exposed floor	Insulation 100 mm, Reinforced concrete 100 mm, cavity 50 mm, chipboard flooring 20 mm	
Boiler efficiency	0.73	
Boiler power	23 kW	
Terminals	6 Radiators: 4 Main, 2 Small	
Thermostat location	Kitchen	
Thermostat Tset point	20 °C	
Scheduling	07:00–9:00 a.m. and 06:00–11:00 p.m.	
Heating system	Boiler plus a single-zone thermostat with a dead-band of 1 °C	

**Table 2:** Analysis of optimization results.

Retrofit Solution	Tset Point	Thermostat Location	POR	Heating Consumption	Energy Cost Post-Retrofit	Retrofit Cost	PB
Raising Tset of existing single zone thermostat	24 °C	Kitchen	3.5%	852 Nm^3^	554 €	-	-
Smart thermostat + 3 TRVs	21 °C	Kitchen + TRVs in each room	3.7%	717 Nm^3^	466 €	375 €	4.2 y
Smart thermostat single zone	21 °C	Bed 1	3.8%	782 Nm^3^	508 €	165 €	3.6 y
**Smart thermostat single zone**	**21 °C**	**Bed 2**	**4.0%**	**745 Nm^3^**	**484 €**	**165 €**	**2.4 y**
Smart thermostat single zone	21 °C	Living R	7.7%	686 Nm^3^	446 €	165 €	1.5 y

**Table 3:** Data statistics of the measured time-series.

Temperature Timeseries	Mean	STD	Min	Max
*T_r_*	18.2 °C	±0.8 °C	15.9 °C	22.9 °C
*T_kitchen_*	19.7 °C	±1.0 °C	16.8 °C	22.2 °C
*T_bedroom_*_2_	17.7 °C	±0.8 °C	14.7 °C	19.6 °C

## Data Availability

The data presented in this study are available on request from the corresponding author. The data are not publicly available due to privacy reasons.
